# Efficient Attack Scheme against SKINNY-64 Based on Algebraic Fault Analysis

**DOI:** 10.3390/e25060908

**Published:** 2023-06-07

**Authors:** Xing Fang, Hongxin Zhang, Xiaotong Cui, Yuanzhen Wang, Linxi Ding

**Affiliations:** 1 School of Electronic Engineering, Beijing University of Posts and Telecommunications, Beijing 100876, China; fancy_t@bupt.edu.cn (X.F.); cuixiaotong@bupt.edu.cn (X.C.); wangyz7@bupt.edu.cn (Y.W.); ding_linxi@163.com (L.D.); 2 Beijing Key Laboratory of Work Safety Intelligent Monitoring, Beijing University of Posts and Telecommunications, Beijing 100876, China

**Keywords:** SKINNY, fault injection, algebraic fault analysis, key residual entropy, single bit

## Abstract

Lightweight block ciphers are normally used in low-power resource-constrained environments, while providing reliable and sufficient security. Therefore, it is important to study the security and reliability of lightweight block ciphers. SKINNY is a new lightweight tweakable block cipher. In this paper, we present an efficient attack scheme for SKINNY-64 based on algebraic fault analysis. The optimal fault injection location is given by analyzing the diffusion of a single-bit fault at different locations during the encryption process. At the same time, by combining the algebraic fault analysis method based on S-box decomposition, the master key can be recovered in an average time of 9 s using one fault. To the best of our knowledge, our proposed attack scheme requires fewer faults, is faster to solve, and has a higher success rate than other existing attack methods.

## 1. Introduction

IoT (Internet of Things) technology has been developing at a rapid pace over the last ten years and is playing an important role in various fields. To protect the data transmitted or processed by such resource-constrained and low-power devices, lightweight block ciphers have emerged and become a research hot spot in cryptography. The more common lightweight block ciphers are SKINNY [1], PRESENT [2], GIFT [3], and LED [4]. Research on these lightweight block ciphers has attracted the attention of many experts and scholars.

Even though the significant goal of a lightweight block cipher is to achieve effective encryption under the condition of limited computational resources, the most crucial and core objective is the security of the cryptography. When we apply a lightweight block cipher to cryptographic devices, we first need to analyze the characteristics of the cryptographic algorithm to ensure the security of the encrypted message. The most common attack methods against cryptographic devices include side-channel analysis and fault analysis. Fault attack, as a powerful attack method, has been one of the pivotal points of research. Fault attack can recover secret information by actively injecting faults into the cryptosystem. For a cryptographic device, the common methods of fault attack mainly include laser fault injection, electromagnetic fault injection, voltage fault injection, temperature fault injection, etc. When a fault is injected into the encryption chip, the encryption algorithm will generate an error in its operation, and thus the faulty ciphertext is obtained as the output of the encryption process, after it is terminated. The structure is not the same between different encryption algorithms. Therefore, when performing a fault attack on an cryptographic device, it is necessary to analyze the encryption algorithms contained in the cryptographic device to provide a specific attack scheme. When a fault occurs, we need to classify and utilize the results generated by the fault; common analysis methods include algebraic fault analysis, differential fault analysis, statistical fault analysis, persistent fault analysis, and algebraic persistent fault analysis. Different analysis methods require different elements such as the number of faults and fault injection locations; so, different experimental schemes need to be designed according to the particular method.

Information security is one of the most critical issues in society that cannot be ignored. If we perform fault analysis and experiments on various lightweight block ciphers from the attacker’s perspective, it is beneficial to the improvement of the security of cryptographic products.

### 1.1. Related Works

SKINNY is a new lightweight tweakable block cipher that was presented to compete with the SIMON family. Currently, the main research methods for SKINNY-64 include differential analysis, differential fault analysis [5,6], impossible differential analysis [7], enhanced persistent fault analysis [8], rectangle attack [9], and algebraic persistent fault analysis [10,11]. The main idea of differential fault analysis is to analyze the XOR differential between the correct and the corresponding faulty ciphertexts to extract the master key. The average number of faults required for the SKINNY-64 block cipher in [5] was 10.6. Impossible differential analysis is a variant of differential cryptanalysis proposed by Knudsen [12] and Biham [13] et al. Its main idea was to discard the wrong keys using differentials that hold with a probability of zero. An impossible differential attack method was proposed in [7] for SKINNY; based on these properties and technique, the new method could break up to 17 rounds of SKINNY-64, but this method did not enable the recovery of the master key. Persistent fault analysis is a new approach to fault analysis proposed in 2018. The main idea of this attack was to inject faults into the lookup table of the S-box and generate errors when the crypto chip calls the lookup table, thus achieving fault injection. The classical persistent fault analysis method is an analysis method for statistical discrepancies in the last round due to the S-box fault, which is solved only for the last round of subkeys. The enhanced persistent fault analysis allows for the analysis and the solution of multiple rounds of subkeys. In total, 1500–1600 fault ciphertexts were required for SKINNY-64 in [8]. Algebraic persistent fault analysis is a combination of algebraic and persistent fault techniques to achieve key recovery by solving algebraic equations. The algebraic persistent fault analysis method used in [10] required a minimum of 10 faulty ciphertexts to achieve recovery of the master key. An overview table of the existing literature on SKINNY-64 is given in Table 1.

An algebraic fault analysis framework for lightweight block ciphers [14] was proposed by Zhang et al. in 2016. The method of counting algebraic equations when using algebraic fault analysis for block ciphers was given in [15]. In previous comparative results of different fault analysis methods against other lightweight block ciphers, algebraic fault analysis was a more efficient attack method. Therefore, this paper adopts algebraic fault analysis as the most fundamental research method for further study. As a masking method, threshold implementation (TI) [16,17] is a kind of side-channel attack countermeasure against first-order differential power analysis (DPA). This method was first proposed by Nikova et al. in 2006. An S-box decomposition scheme was proposed in [16] that could transform the cubic S-box used in lightweight block ciphers into two quadratic S-boxes. The specific steps for the S-box decomposition of the GIFT block cipher were given in [18]. Ref. [11] combined these two methods to propose an algebraic persistent fault analysis, which achieved efficient experimental results. Inspired by this approach, we use the method of S-box decomposition to optimize the algebraic equation expressions of the S-box and thus improve the efficiency of the solver.

Due to the structural characteristics of the SKINNY block cipher, the diffusion paths of a single fault in different locations of the cipher are varied, and analyzing the impact of different locations on fault diffusion can further improve the efficiency of the attack.

In order to further reduce the number of faults required in the attack on SKINNY-64 and improve the solving efficiency, we need to synthesize several methods, and we propose a more optimal fault injection scheme in this paper.

### 1.2. Contributions

In this paper, we perform differential trace analysis and algebraic fault analysis based on S-box decomposition of the SKINNY-64 block cipher. Through analysis and experiments, choosing the appropriate location of fault injection, the recovery of the master key is possible within 10 s using one fault. The main features can be summarized as follows:

- By analyzing the structure and round function of SKINNY-64, we express the encryption process algebraically. An optimized algebraic equation representation for the S-box is proposed for SKINNY-64 using the S-box decomposition technique. An improved algebraic fault analysis method for SKINNY-64 is implemented based on the above information.
- Due to the characteristics of SKINNY-64, when the fault is in different rows of the same round, the fault diffusion effect is different. An efficient fault injection scheme is given by analyzing the diffusion of a single fault at different locations in the 27th and 28th rounds.
- The two algebraic fault analysis methods are compared by several simulation experiments. The appropriate fault injection location and fault utilization method are given by comparing the solving success rate and the average solving time within the specified time.

The remainder of this paper is organized as follows. We give the related preliminaries in Section 2. In this section, we first give a general description of SKINNY-64; then, an algebraic fault analysis method for SKINNY-64 is briefly introduced. In Section 3, we analyze the effect of the fault diffusion for different fault locations. An efficient fault injection scheme is given by analysis. We provide our experimental results for SKINNY-64 in Section 4. Section 5 provides a discussion, followed by a conclusion and future work in Section 6.

## 2. Preliminaries

In this section, we briefly describe the SKINNY-64 specification. Next, we give the expansion scheme of the subkeys and show the subkeys of the last rounds in graphical form. Finally, we give an improved scheme with algebraic fault analysis based on S-box decomposition against SKINNY-64.

### 2.1. General Description of SKINNY-64

**Figure 1:**
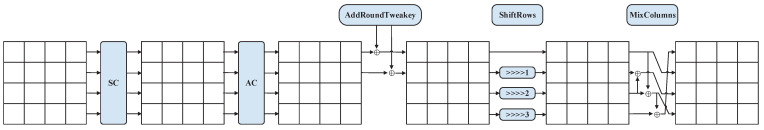
Schematic diagram of the SKINNY-64 round function.

SKINNY follows a Substitution-Permutation-Network (SPN) structure. The specification of SKINNY describes iterative rounds consisting of a total of five suboperations: SubCells (SC), AddConstants (AC), AddRoundTweaks (ART), ShiftRows (SR), and MixColumns (MC). In this paper, we focus on analyzing SKINNY-64-64. The number of rounds for SKINNY-64-64 is 32. Figure 1 presents a schematic diagram of the SKINNY-64 with the round function.

- SubCells

The SubCells operation is a nonlinear bijective transformation in the encryption process. Table 2 gives the S-box lookup table in form of the hexadecimal notation.

To prepare the algebraic analysis, let the input of the S-box be $x_{3}\|x_{2}\left\|x_{1}\right\|x_{0}$ and the output of the S-box be $y_{3}\|y_{2}\left\|y_{1}\right\|y_{0}$. According to the relevant information provided in [1], the algebraic input–output relationship regarding the S-box can be expressed by the following equation.

(1) $$\begin{array}{cc} y_{0} & =x_{1}+x_{2}+x_{3}+x_{0}x_{1}+x_{0}x_{2}+x_{0}x_{3}+x_{1}x_{3}+x_{0}x_{1}x_{2}+x_{1}x_{2}x_{3},\\ y_{1} & =x_{0}+x_{3}+x_{0}x_{1}+x_{1}x_{2}+x_{1}x_{3}+x_{2}x_{3}+x_{1}x_{2}x_{3},\\ y_{2} & =1+x_{1}+x_{2}+x_{3}+x_{1}x_{2},\\ y_{3} & =1+x_{0}+x_{2}+x_{3}+x_{2}x_{3}. \end{array}$$

- Addconstants

The Addconstants is an operation of SKINNY that adds the round constants with the internal state. The constants are generated using a six-bit affine linear feedback shift register (LFSR), whose state is updated by the following definition,

(2) $$\left(rc_{5},rc_{4},rc_{3},rc_{2},rc_{1},rc_{0}\right)\leftarrow \left(rc_{4},rc_{3},rc_{2},rc_{1},rc_{5}⊕rc_{4}⊕1\right).$$

- ShiftRows

The ShiftRows operation performs a cell-wise right-rotation of 0, 1, 2, and 3 cells for the first, second, third, and fourth rows of the internal state. A permutation P is applied on the cells positions of the cipher internal state cell array: for all 0≤i≤15, the operation can be represented as P=[0,1,2,3,7,4,5,6,10,11,8,9,13,14,15,12].

- MixColumns

The MixColumns operation of SKINNY-64 multiplies the internal state with a matrix *M*. The matrix *M* is given as

(3) $$\left[\begin{array}{cccc} 1 & 0 & 1 & 1\\ 1 & 0 & 0 & 0\\ 0 & 1 & 1 & 0\\ 1 & 0 & 1 & 0 \end{array}\right].$$

### 2.2. Subkeys of SKINNY-64

The AddRoundTweakey operation of the cipher is that the first and second rows of all tweaked arrays are extracted and bitwise exclusive-or to the cipher’s internal state. For the SKINNY-64, a permutation PT is applied on the cell positions of all the tweaked arrays.

(4) PT=[9,15,8,13,10,14,12,11,0,1,2,3,4,5,6,7]

**Figure 2:**
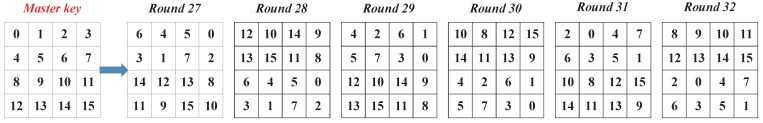
The subkeys of SKINNY-64.

According to the provided subkeys generation scheme, the subkeys of the last rounds are shown in Figure 2.

### 2.3. Algebraic Fault Analysis against SKINNY-64

Algebraic fault analysis (AFA), combining algebraic cryptanalysis with fault analysis, was proposed by Courtois et al. in [19]. A machine solver can be used to automatically recover the secret key. In literature [14], Zhang et al. proposed a framework for the analysis and evaluation of algebraic fault attacks on lightweight block ciphers.

For the process of building algebraic equations for the algebraic fault analysis of lightweight block ciphers, the complicated part is the S-box substitution operation. SubCells, as the only nonlinear component in the SKINNY-64, are represented using a larger number of variables and CNF equations.

- Representing the SubCells

The input–output relationship of SubCells is shown in Equation (1). In this paper, we use the CryptoMiniSAT solver for solving algebraic equations. The Subcells per round can be represented using 192 variables and 496 CNF equations.

- Representing the AddConstants, AddRoundTweaks, ShiftRows, and MixColumns

For other operations, we represent the round constant with six variables, and each round of AddConstants can be represented with 64 variables and 64 CNF equations. Each round of AddRoundTweaks can be represented with 128 variables and 128 CNF equations. Each round of ShiftRows can be represented using 64 variables and 64 CNF equations, and the MixColumns can be represented with 64 variables and 64 CNF equations.

In summary, each round of encryption can be represented with 518 variables and 816 CNF equations.

In our previous research, an S-box decomposition scheme was proposed to decompose a cubic S-box into two quadratic S-boxes, thus reducing the number of quadratic and cubic variables in the algebraic equations. By rewriting the algebraic equations of the S-box, the speedup and the reduction of the number of faulty samples can be achieved. Table 3 gives the two quadratic S-boxes of the SKINNY algorithm after S-box decomposition.

The algebraic equations for two quadratic S-boxes are represented as Equations (5) and (6), respectively.

(5) $$\begin{array}{cc} F(w,z,y,x) & =(f_{3},f_{2},f_{1},f_{0})\\ f_{0} & =x+z+xw+w\\ f_{1} & =y\\ f_{2} & =y+yz+z+w\\ f_{3} & =z \end{array}$$

(6) $$\begin{array}{cc} H(w,z,y,x) & =(h_{3},h_{2},h_{1},h_{0})\\ h_{0} & =y+yz\\ h_{1} & =x+xy+w\\ h_{2} & =1+z\\ h_{3} & =1+x \end{array}.$$

The new S-box algebraic equations can be represented by 192 variables and 320 CNF equations per round. We use fewer CNF equations to represent the same encrypted information and reduce the use of higher-order variables in the equations, which can effectively improve the efficiency of the solver.

In this paper, we assume that an attacker can inject a single-bit fault at exactly one location of the encryption process, and the attacker can obtain the fault injection information and the faulty ciphertext. Therefore, we can create new algebraic equations for the incorrect encryption process according to the method provided in [14]. After we have determined the method of fault utilization, we need to select the optimal fault injection location. Since there is a ShiftRows operation in the SKINNY-64, and the shift value is different for each row, the diffusion effect is very variable when the fault appears in different rows. Therefore, it is necessary to analyze the impact of all fault locations.

## 3. Analysis of the Efficient Location for Fault Injection

The basic principle of the fault attack is to recover the master key by injecting faults in the encryption process, at the same time, associating the correct ciphertext, faulty ciphertexts, and other information by establishing the relationship between the faulty and correct encryption. When performing a fault attack, the location of the fault is important. If the round of the fault is shallow, the fault does not spread sufficiently, and the number of required faults is elevated. If the round of fault is too deep, it leads to information redundancy and is not conducive to key recovery.

**Figure 3:**
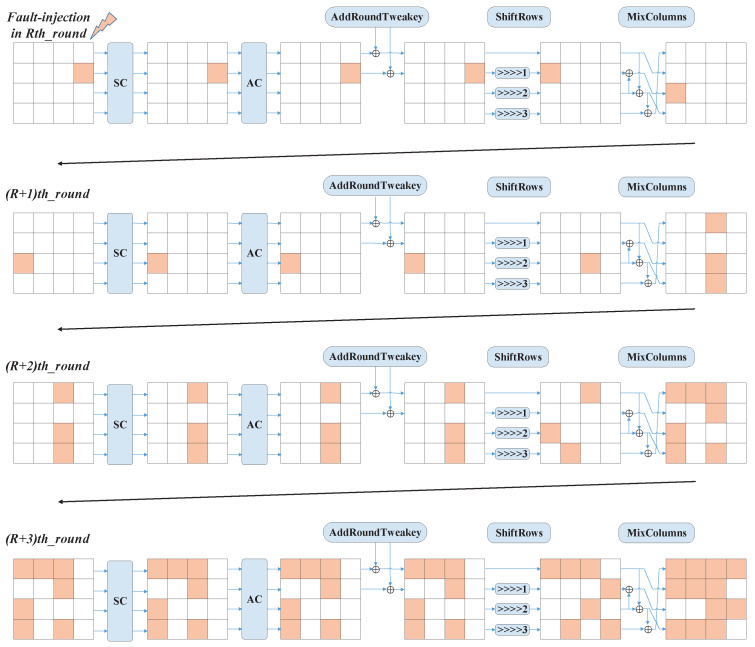
The diffusion diagram of SKINNY-64 with the fault injected in the beginning of *R*-th round.

Figure 3 gives the fault propagation procedure of SKINNY-64. In the figure, we inject a fault in the seventh cell at the beginning of round *R*. After four rounds of cryptographic operations, the fault spreads to different cells. The SubCells, Addconstant, and AddRoundKey operations do not affect the diffusion of the faults. The ShiftRows operation allows the faults to move to other columns by row shifting. The MixColumns operation is the most important operation that causes fault spreading. Due to the structural characteristics of the SKINNY-64, the rules of each row are different; so, the impact of the fault diffusion will be different. At the same time, since only the first and second rows of each round perform the AddRoundTweakey operation, the effect of the fault on the round key should also be considered when analyzing the fault propagation.

**Figure 4:**
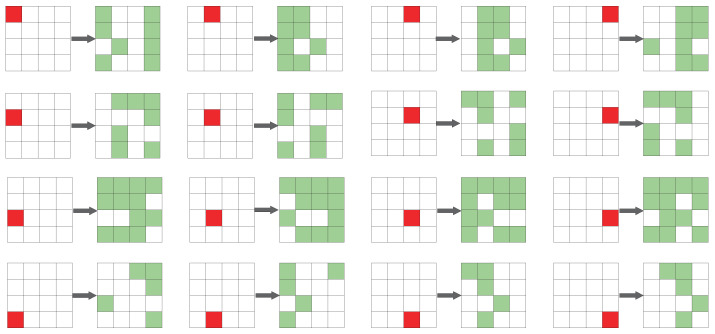
Schematic diagram of different locations of the fault after three rounds of diffusion (The red box represents the initial fault, and the green boxes represent the diffused faults after three rounds).

Therefore, we injected faults into 16 possible locations, and the schematic diagram after three rounds of fault propagation is shown in Figure 4. All these fault patterns are distinct. It is very interesting to note that when the faults are in the same row, the patterns resulting from the four faults are similar, and the same pattern can be obtained by shifting. When the faults are in different rows, they have very different patterns; in other words, the effect of the fault propagation is different. When the fault is injected into the first row, after three rounds, the fault spreads to eight cells; meanwhile, when the fault is injected into the second, third, and fourth rows, the fault spreads to seven, twelve, and five cells, respectively. The patterns remain the same up to the input of the RowShifts operation at the later round.

According to the above analysis, firstly, we inject the fault into the 29th round. The single fault will obtain the patterns given in Figure 4 after the 29th, 30th, and 31st rounds of encryption operations. At the same time, this pattern will be maintained until the 32nd round of AddRoundTweaks.

**Figure 5:**
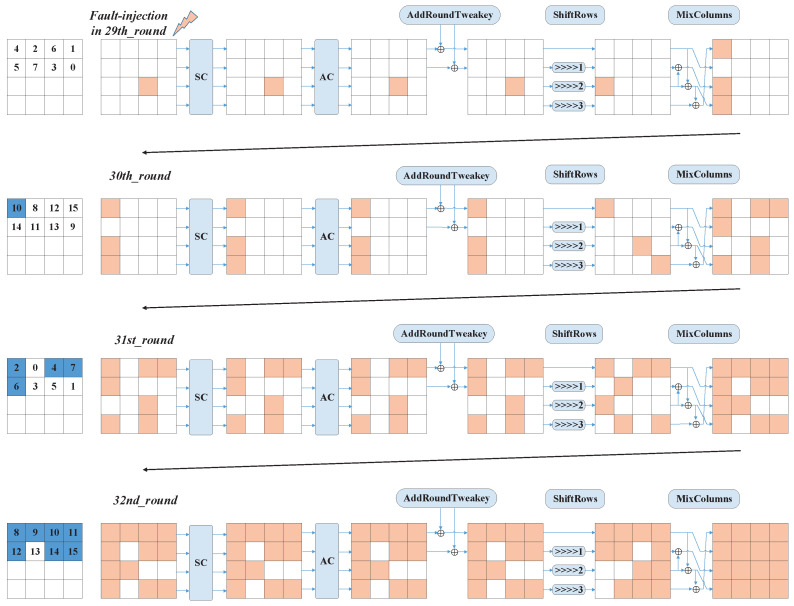
The schematic diagram for the index of affected keys after four rounds (The blue box indicates the index of the affected keys, and the orange box indicates the propagation path of the fault).

Using the example of a fault injected into the third column of the third row of round 29, Figure 5 gives the index of the affected keys after four rounds of diffusion of a single fault. At the 29th round, no subkey is affected because the fault occurred in the third row, which was not involved in the AddRoundTweaks operation. After the 29th round of MixColumns, the fault spread to rows 1, 3, and 4. At the 30th round, the fault in the first column of the first row was involved in the AddRoundTweakey operation, and its corresponding key index was 10. Similarly, when the fault spread to the last round, the affected key index array was [2,4,6,7,8,9,10,11,12,14,15].

Similarly, we analyzed 16 different fault locations for the 29th round and provide the affected key index arrays separately in Table 4.

From Table 4, it can be noticed that the largest number of affected key indexes was obtained when the fault was injected into four locations in the third row of the 29th round. In the case where the number of faults was one, the more keys that were affected by the fault, the more information was generated for the key recovery, which was more favorable for recovering the master key. For the key indexes that were not affected by the fault, the solver used a violent search method for the unaffected key indexes, which was very time-consuming.

**Figure 6:**
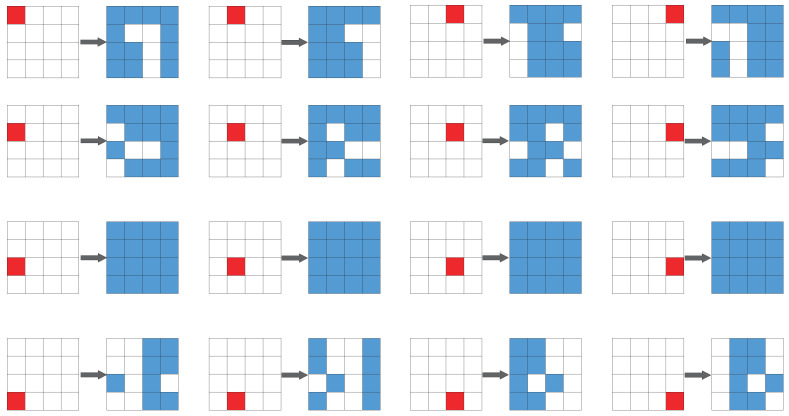
Schematic diagram of different locations of the fault after four rounds of diffusion (The red box represents the initial fault, and the blue boxes represent the diffused faults after four rounds).

**Figure 7:**
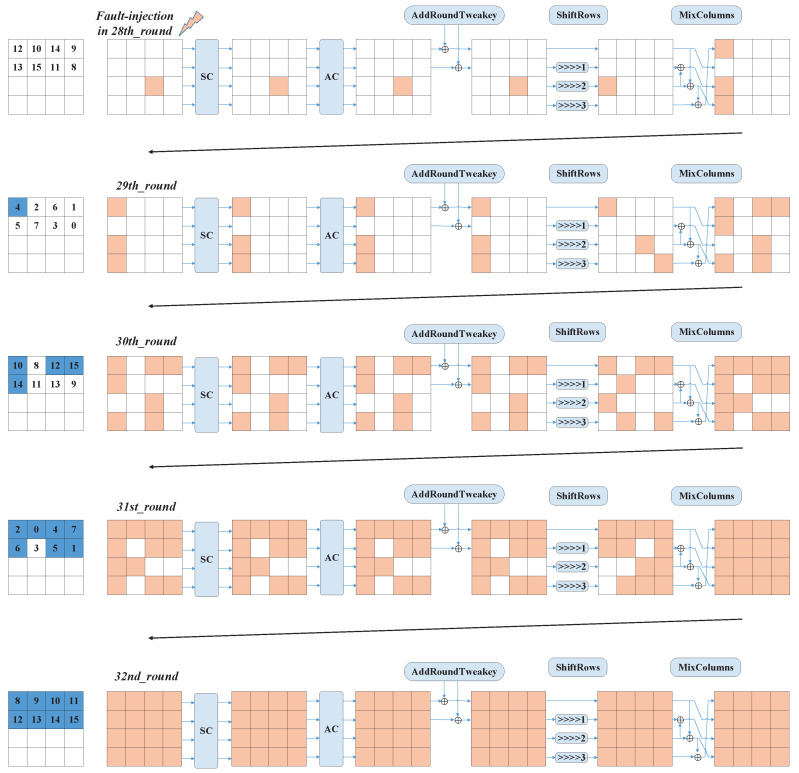
The schematic diagram for the index of affected keys after five rounds (The blue box indicates the index of the affected keys, and the orange box indicates the propagation path of the fault).

From the above analysis, we can find that the depth of the fault was not deep enough to cause sufficient diffusion using one fault. Therefore, we injected the fault into the beginning position of the 28th round. According to the different locations of the fault, we give a schematic diagram of the 16 types of fault diffusion in Figure 6. As we can see in Figure 6, when the fault was injected into the third row at the beginning of the 28th round, the fault affected all positions after four rounds of diffusion. Similarly, we still used the example of a fault injection into the third column of the third row. From Figure 7, we see that the arrays of the affected key indexes after five rounds were [4] + [10,12,14,15] + [0,1,2,4,5,6,7] + [8,9,10,11,12,13,14,15], merged as [0,1,2,4,5,6,7,8,9,10,11,12,13,14,15]. After five rounds of encryption, the fault injected into the 10th cell at the beginning of the 28th round was fully diffused.

We analyzed the other 15 positions at the beginning of the 28th round and provide the results in Table 5. From Table 4, we can find that the number of unaffected key indexes was the lowest when the fault location was in SBox_8, SBox_9, SBox_10, and SBox_11. These locations were all in the third row at the beginning of the 28th round.

As mentioned above, the diffusion effect of the fault was different when the fault occurred on different rows, and the affected key index arrays were also different. Next, we used software to simulate fault attacks and analyzed the efficiency of the key recovery when the fault was in different scenarios.

## 4. Simulation Experiments and Results

To verify the effectiveness of the proposed attack scheme in reducing the number of faults and the time of key recovery, we conducted multiple sets of randomized experiments and statistically analyzed the average solving time and solving success rate for different scenarios. In our experiments, we simulated the fault injection experiment via Python. We implemented the experiments on a PC that had 16 GB memory and Intel(R) Core(TM) i5-9500 CPU at 3 GHz. The operating system was a 64-bit Windows 10. After generating the algebraic equations file for the solver using the Python script, we completed the solution of the algebraic equations using the CryptoMiniSAT solver under Ubuntu 18.04.5.

Algorithm 1 was used to implement the encryption of the SKINNY-64 block cipher. The inputs of the algorithm were *P* (plaintext) and *K* (master keys), and the output was the correct ciphertext *C*, where MC, SR, and RC represent the MixColumn, ShiftRow, and round constants, respectively. We obtained the correct encryption result in the output, which was used in the algebraic fault analysis.
**Algorithm 1:** The encryption of SKINNY-64.
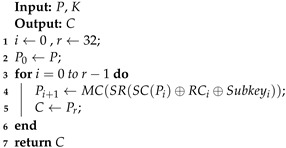


Algorithm 2 was used for the process of fault injection in the SKINNY-64 block cipher. The inputs of the algorithm were *P* (plaintext), *K* (master keys), and $R_{fault}$, and the output was the fault ciphertext $C^{*}$. Due to the innovation of the fault injection technology, we can achieve the precise injection of fault using methods such as laser fault injection to achieve a single-bit fault. Here, we used simulated fault injection to obtain the fault ciphertext.

In Algorithm 3, we give a pseudocode for an efficient algebraic fault attack scheme against SKINNY-64. The inputs of the algorithm were *P*, $R_{fault}$, and *N*, which represented the plaintext, the number of fault rounds, and the number of instances, respectively. In Line #1, we used algebraic equations based on S-box decomposition instead of the original algebraic expressions. Similarly, we used the new S-box expressions when building the equations for faulty encryption in Line #2. In Line #3, we used the CryptoMiniSAT to recover the master keys.
**Algorithm 2:** Fault injection to SKINNY-64.
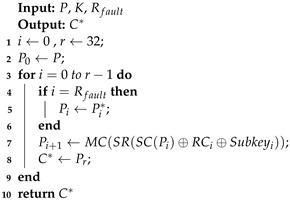


**Algorithm 3:** Efficient algebraic fault attack scheme against SKINNY-64.

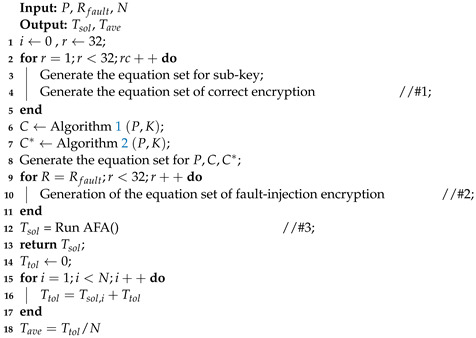



We generated 50 random instances for each scenario and set the solver solving time to 1 hour, after which the attack was considered to have failed. The average solving time and the success rate of solving for different scenarios are given in Table 6. It should be noted that the “-” symbol appearing in Table 6 means that all 50 samples could not recover the master key within a specific time; so, the average solving time had no definite value.

**Figure 8:**
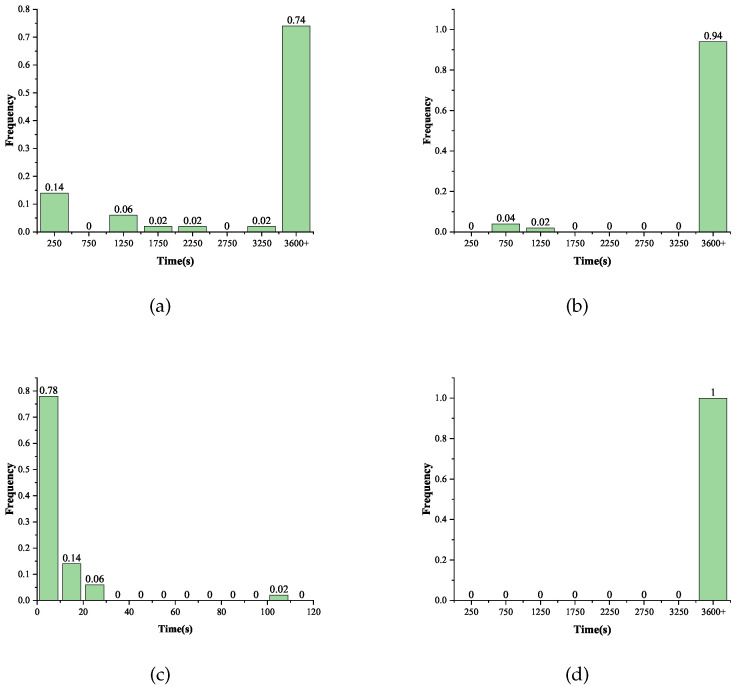
Distribution of the solving time under different scenarios in the 28th round. (**a**) Locations = [0,1,2,3]; (**b**) Locations = [4,5,6,7]; (**c**) Locations = [8,9,10,11]; (**d**) Locations = [12,13,14,15].

The experimental results indicated that when the fault was injected into the 29th round, the recovery of the master key was not achieved for one fault within the specified time, regardless of the location of the fault injection. Analyzed from the perspective of the algebraic representation of the S-box, no key recovery could be completed within the specified time using the original S-box algebraic equations under the premise of using one fault. When the fault was injected into the first row at the beginning of the 28th round, the success rate of the attack in 50 instances was 26%, and the average solving time was 959.2 s. When the fault was injected into the second row, the success rate was 6%, and the average solving time was 1007.0s. The most interesting experimental result was that when the fault was injected into the third row of the 28th round, the success rate of the solving within the specified time was 100%, and the average solving time was 9.0 s. Meanwhile, when the fault was injected into the fourth row of the 28th round, none of the attacks could be completed. The histogram of the frequency distribution of the solving time for different scenarios in the 28th round is given in Figure 8. From the experimental results, it can be seen that the use of the S-box decomposition method proposed in this paper made it possible to complete the algebraic fault analysis of the SKINNY-64 with a single fault, while the attack could not be achieved in a specific time using the original S-box. We gave the optimal fault injection location by analyzing the diffusion effect of different locations, and the experimental results were consistent with the analysis.

**Figure 9:**
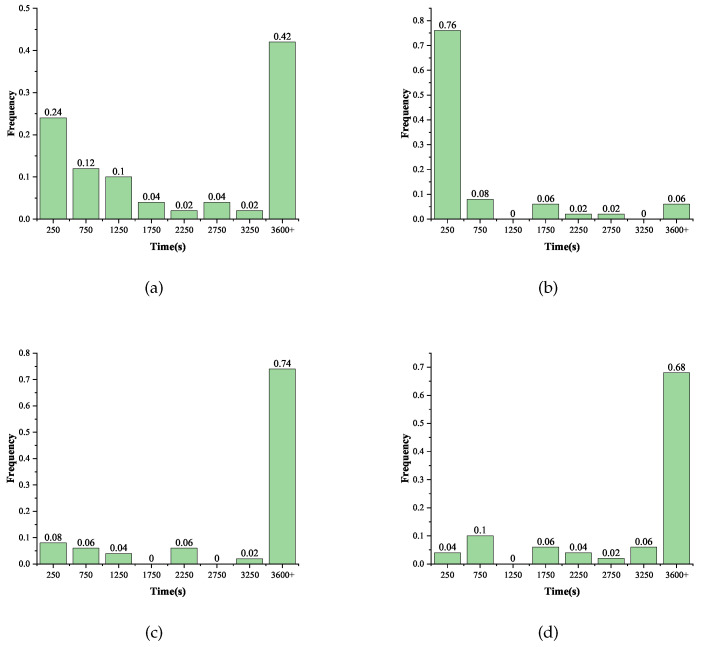
Distribution of the solving time under different scenarios in the 27th round. (**a**) Locations = [0,1,2,3]; (**b**) Locations = [4,5,6,7]; (**c**) Locations = [8,9,10,11]; (**d**) Locations = [12,13,14,15].

In the same way, we continued to deepen the fault injection depth. When the fault was injected into the first row at the beginning of the 27th round, the success rate of the attack in 50 instances was 58%, and the average solving time was 956.8 s. When the fault is injected into the second row, the success rate was 94%, and the average solving time was 373.8 s. Unlike the fault injection into the 28th round, when the fault was injected into the third row of the 27th round, its solving success rate decreased to 26%, and the average solving time increased from 9 s to 1236.6 s. This could be caused by information redundancy due to the faults diffusing too quickly. Meanwhile, when the fault was injected into the fourth row of the 27th round, the success rate was 32%, and the average solving time was 1594.0 s. Similarly, recovery of the master key within one hour could not be achieved in 27 rounds using one fault without the S-box decomposition method. The histogram of the frequency distribution of the solving time for different scenarios in the 27th round is given in Figure 9.

We tried to perform experiments in deeper rounds, and the experimental results showed that the key recovery could not be completed in the specified time under this attack model.

## 5. Discussion

By performing differential trace analysis and improved algebraic fault analysis on SKINNY, we found that the recovery of the master keys could be achieved using one fault. This attack scheme was efficient and achievable. When the fault was injected in the 28th round, the best fault injection point was the third row. When the fault was injected in the 27th round, the best fault injection point was the second row. Choosing the appropriate fault injection location could effectively reduce the number of faults required and improve the solving efficiency. At the same time, improving the representation of the algebraic equations could also improve the success rate of the attack and reduce the number of faults required for the attack. The attack scheme proposed in this paper has the advantages of a lower number of faults and a faster solving speed compared with those proposed in the existing literature. A comparison chart between the different approaches is given in Table 7.

By comparing the literature in Table 7, we can find that the fault injection locations of the DFA method proposed in [5] and the method used in this paper were both in the 28th round. The number of faults used in [5] was 10.6; meanwhile, only one fault was needed in this paper to complete the recovery of the master key. There was little difference in time between the two methods, and both completed the attack in a relatively short time. Ref. [10] and ref. [8] are studies based on persistent fault analysis. First, a fault was injected into the S-box, causing the cryptographic device to fail during operation. The attacker performed key recovery based on the generated faulty ciphertexts and related fault information. The algebraic persistent fault analysis proposed in [10] for SKINNY-64 required a minimum of 10 fault ciphertexts to recover the master key, and from the experimental results, the average solving time reached $3.7\times 10^{5}$. The enhanced persistent fault analysis proposed in [8] for SKINNY-64 required a number of faulty ciphertexts between 1500 and 1600, with an average solving time of 0.03s. Although this method had a shorter solving time, it required a larger number of faulty ciphertexts. Therefore, after trading off various elements, it is concluded that the method proposed in this paper is more efficient and implementable.

## 6. Conclusions and Future Work

This paper investigated the SKINNY-64 block cipher using algebraic fault analysis. Compared with the classical algebraic fault analysis framework, the S-box decomposition technique and the differential path analysis method were introduced in this paper, and the optimal fault injection model was given according to the characteristics of the algorithm itself. The S-box decomposition technique reduces the number of algebraic equations to some extent, expresses the same information using fewer CNF equations, and improves the solving time of the Solver. The more obvious experimental conclusion is that the use of the S-box decomposition technique enables the recovery of one fault for all keys in the context of the same fault attack. As for the analysis of differential paths, choosing the most suitable fault injection point can effectively improve the fault injection efficiency. The structural properties of the algorithm lead to differences in the robustness of different locations. The optimal fault injection point was analyzed by analyzing how the subkey index was affected when the fault was at different locations. With the popularity of lightweight block ciphers, many different lightweight block ciphers have appeared on the market. They have similar but different structures. In order to better implement attack testing and protection, a targeted analysis of the different ciphers is needed. The structure of the LED-64 is similar to that of the SKINNY-64 in that they both contain RowShifts operation. The proposed method based on this paper is also applicable to the LED-64, and we will study the LED block cipher and give an efficient algebraic fault analysis method for it in future work.

The two main forms contained in lightweight cryptographic algorithms are SPN-type and Feistel-type. Currently, we have only conducted related research for SPN-type lightweight block ciphers. In future work, we can use the analysis method proposed in this paper to investigate cryptographic algorithms with Feistel-type structures.

## Figures and Tables

**Table 1:** Overview of the existing literature on SKINNY-64.

Methods	Reference	Minimum Number of Faults	Recover Master Key	Year Published
DFA	[5]	10.6	Yes	2018
IDA	[7]	-	No	2017
EPFA	[8]	1500–1600	Yes	2021
APFA	[10]	10	Yes	2022
APFA	[11]	10	Yes	2022
RA	[9]	-	No	2021

**Table 2:** S-box lookup table used in SKINNY-64.

X	0	1	2	3	4	5	6	7	8	9	a	b	c	d	e	f
S[X]	c	6	9	0	1	a	2	b	3	8	5	d	4	e	7	f

**Table 3:** The decomposed S-boxes.

X	0	1	2	3	4	5	6	7	8	9	a	b	c	d	e	f
F[X]	0	1	6	7	d	c	f	e	5	4	3	2	9	8	b	a
H[X]	c	6	d	5	8	3	9	0	e	4	f	7	a	1	b	2

**Table 4:** The index distribution of the affected keys under different fault locations in the 29th round.

Location	Index of Affected Keys	Index of Unaffected Keys
SBox_0	[2,4,6,7,8,10,11,12,14,15]	[0,1,3,5,9,13]
SBox_1	[0,2,3,8,9,11,12,13]	[1,4,5,6,7,10,14,15]
SBox_2	[0,4,5,6,9,10,12,13,14]	[1,2,3,7,8,11,15]
SBox_3	[1,4,7,9,10,11,14,15]	[0,2,3,5,6,8,12,13]
SBox_4	[5,7,9,10,11,15]	[0,1,2,3,4,6,8,12,13,14]
SBox_5	[2,7,8,10,11,12]	[0,1,3,4,5,6,9,13,14,15]
SBox_6	[0,3,8,9,11,13]	[1,2,4,5,6,7,10,12,14,15]
SBox_7	[0,4,8,9,10,14]	[1,2,3,5,6,7,11,12,13,15]
SBox_8	[0,2,4,5,8,9,10,11,12,13,14]	[1,3,6,7,15]
SBox_9	[0,1,4,7,8,9,10,11,13,14,15]	[2,3,5,6,12]
SBox_10	[2,4,6,7,8,9,10,11,12,14,15]	[0,1,3,5,13]
SBox_11	[0,2,3,7,8,9,10,11,12,13,15]	[1,4,5,6,14]
SBox_12	[1,7,10,11,15]	[0,2,3,4,5,6,8,9,12,13,14]
SBox_13	[2,6,8,10,11,12]	[0,1,3,4,5,7,9,13,14,15]
SBox_14	[0,3,8,9,13]	[1,2,4,5,6,7,10,11,12,14,15]
SBox_15	[4,5,9,10,12,14]	[0,1,2,3,6,7,8,11,13,15]

**Table 5:** The index distribution of the affected keys under different fault locations in the 28th round.

Location	Index of Affected Keys	Index of Unaffected Keys
SBox_0	[1,2,4,5,6,7,8,9,10,11,12,14,15]	[0,3,13]
SBox_1	[0,2,3,6,7,8,9,10,11,12,13]	[1,4,5,14,15]
SBox_2	[0,3,4,5,6,8,9,10,11,13,14]	[1,2,7,12,15]
SBox_3	[0,1,4,5,7,8,9,10,11,12,14,15]	[2,3,6,13]
SBox_4	[0,1,4,7,8,9,10,11,13,14,15]	[2,3,5,6,12]
SBox_5	[2,6,7,8,9,10,11,12,14,15]	[0,1,3,4,5,13]
SBox_6	[0,2,3,7,8,9,10,11,12,13,15]	[1,4,5,6,14]
SBox_7	[0,2,4,5,8,9,10,11,12,13,14]	[1,3,6,7,15]
SBox_8	[0,2,3,4,5,6,7,8,9,10,11,12,13,14,15]	[1]
SBox_9	[0,1,2,3,4,5,7,8,9,10,11,12,13,14,15]	[6]
SBox_10	[0,1,2,4,5,6,7,8,9,10,11,12,13,14,15]	[3]
SBox_11	[0,1,2,3,4,6,7,8,9,10,11,12,13,14,15]	[5]
SBox_12	[1,4,7,9,10,11,14,15]	[0,2,3,5,6,8,12,13]
SBox_13	[2,4,6,7,8,10,11,12,15]	[0,1,3,5,9,13,14]
SBox_14	[0,2,3,8,9,11,12,13]	[1,4,5,6,7,10,14,15]
SBox_15	[0,4,5,6,9,10,12,13,14]	[1,2,3,7,8,11,15]

**Table 6:** The results of the attack against SKINNY-64 under different scenarios.

Round	Location	$T_{\mathrm{ave}}$ (Seconds) of Original S_Box	$T_{\mathrm{ave}}$ (Seconds) of New S_Boxes	Success Rate of Original S_Box	Success Rate of New S_boxes
29	[0,1,2,3]	-	-	0%	0%
29	[4,5,6,7]	-	-	0%	0%
29	[8,9,10,11]	-	-	0%	0%
29	[12,13,14,15]	-	-	0%	0%
28	[0,1,2,3]	-	959.2	0%	26%
28	[4,5,6,7]	-	1007.0	0%	6%
28	[8,9,10,11]	-	9.0	0%	100%
28	[12,13,14,15]	-	-	0%	0%
27	[0,1,2,3]	-	956.8	0%	58%
27	[4,5,6,7]	-	373.8	0%	94%
27	[8,9,10,11]	-	1236.6	0%	26%
27	[12,13,14,15]	-	1594.0	0%	32%

**Table 7:** Comparison with existing fault analyses of SKINNY-64.

Methods	Reference	Location of Fault	Minimum Number of Faults	Average Attack Time(s)
DFA	[5]	28th round	10.6	0.28
APFA	[10]	S-box table	10	$3.7\times 10^{5}$
EPFA	[8]	S-box table	1500–1600	0.03
AFA	this paper	28th round	1	9
AFA	this paper	27th round	1	373.8

## Data Availability

https://github.com/fancy-t/skinny_AFA_new_sbox_optional, (accessed on 22 May 2023) offers the relevant data.
